# Charting the future of kidney care in Brazil: insights and evolution through the Brazilian Dialysis Survey

**DOI:** 10.1590/2175-8239-JBN-2024-E006en

**Published:** 2024-05-24

**Authors:** Roberto Pecoits

**Affiliations:** 1 DOPPS Program Area, Arbor Research Collaborative for Health. Ann Arbor, MI, USA.

The importance of data cannot be overstated in addressing the increasingly complex challenges of kidney care. Observational data informs policy, guides clinical decisions, and most importantly, shapes the future of patient care. The Brazilian Dialysis Survey (BDS), spanning more than two decades, stands as a testament to the Brazilian Society of Nephrology (BSN)’s commitment to understanding and improving the landscape of kidney care. As we reflect on the contributions of this initiative, it’s crucial to celebrate the progress it has enabled while acknowledging the areas that need further development.

## A Legacy of Insight

Since 1999, the BDS has served as a cornerstone for nephrology research and practice in Brazil. By systematically capturing and analyzing data from nephrology clinics across the country, the census has provided invaluable insights into trends, practice patterns, and disparities in kidney care. Over the years, these data have informed the nephrology (and the broader healthcare) community, as well as policy makers on the dialysis landscape. The BDS has likely increasingly influenced clinical and policy decisions aimed at improving patient care. The commitment of participating clinics to voluntarily conduct this census, spearheaded by the BSN, underscores the collective dedication to developing kidney care in Brazil. The latest report, based on the 2022 survey, is included in this edition of the Brazilian Journal of Nephrology^(1)^.

## The Power of Collective Data

The data collected over the years by the BDS offer a panoramic view of the evolution of dialysis care, including patient demographics and distribution of treatment modalities. This longitudinal perspective is crucial for identifying shifts in disease prevalence, changes in treatment efficacy, and emerging challenges in patient management. Such insights are instrumental for shaping targeted interventions, allocating resources effectively, and forecasting future needs within the nephrology community. The 2022 BDS highlights a continuation of the historical trend in the increase in both the number and prevalence rate of patients on chronic dialysis, with an impressive growth of hemodiafiltration (HDF) as a modality, currently matching the numbers of peritoneal dialysis (4.6% on HDF and 4.7% on peritoneal dialysis). It is unfortunate, however, that the utilization of HDF is limited to patients with private insurance. Only a very small percentage (1.3%) of dialysis patients were not vaccinated against COVID-19, a positive sign of the national strategy implemented by the Brazilian national public health system. The overall crude annual mortality rate was 17.1%, indicating a decrease from previous years, possibly due to the end of the COVID-19 pandemic.

## Voices of Critique: the Call for Evolution

Despite its substantial contributions, the BDS is not without its limitations. A critical aspect that warrants attention is the methodology of data collection. The framework used historically and in the 2022 edition of the BDS primarily captures clinic-level data and neglects the granularity and richness of individual patient-level information. This limits the depth of analysis possible, potentially obscuring nuanced trends and patterns that could lead to more personalized and effective care strategies.

Moreover, the voluntary nature of participation in the census introduces a significant bias, as data are collected from a subset of clinics that may not represent the entire spectrum of nephrology care across Brazil. This selective participation limits the generalizability of the findings and may lead to an incomplete picture of the nephrology landscape, affecting the accuracy of assessments and the formulation of national health policies.

## Embracing Change: Insights and Opportunities from the 2023 Census

The most recent 2023 census, already available through the BSN website^(2)^, marks a pivotal moment in the evolution of the BDS. The 2023 edition census showcases a significant leap forward, reflecting the highest participation of clinics in recent years: 37.5% of the 886 active clinics caring for more than 60 thousand dialysis patients. This surge in contributions is a testament to the BSN’s dedicated efforts to better reach clinics. By leveraging academic nephrology leagues, fostering collaboration with large dialysis organizations, and facilitating direct engagements between regional representatives and the BSN, a broader, more inclusive data landscape has been created. Another important innovation introduced in the 2023 BDS is the use of a random sample of responders as a source for prevalence and incidence calculations. The fact that this approach yielded similar results compared to historical figures reassures the accuracy of the data captured over the years.

Moreover, the involvement of a younger generation of kidney epidemiologists in the leadership of the census and registries, and the expansion of research opportunities reflected in a growing number of ancillary publications using the BDS as a data source has infused the project with fresh perspectives and innovative approaches. This diversification heralds a new era of research and analysis in Brazilian nephrology, promising richer insights and a more dynamic understanding of kidney health across the country.

## A Path Forward: Future Recommendations

As a Brazilian expatriate deeply interested in the epidemiology of CKD and in kidney care, I am tempted to list some insights to further increase the richness and utility of the BDS:

1. **Forge partnerships with the Brazilian public healthcare system with the aim of diversifying data sources**: A key initiative is to align and collaborate with Brazil’s public healthcare system to gain access to public databases and integrate them with the rich epidemiologic information of the BDS and disease registries. Following models such as the United States Renal Data System (USRDS), which integrates data from the Centers for Medicare and Medicaid Services (CMS), the Veteran Affairs system, and commercial claims databases, such partnerships could significantly enrich the information provided by the Brazilian Nephrology Census. Leveraging other kidney care databases would allow for a more comprehensive collection of patient-level data across the healthcare spectrum, facilitating deeper insights into kidney disease trends, treatment outcomes, and healthcare disparities. One example of such enrichment is the 2023 USRDS Annual Data Report^(3)^.
2. **Embrace technology and innovation**: Digital platforms, artificial intelligence, and data analytics tools offer opportunities to revolutionize the way data is gathered, analyzed, and shared within the nephrology community. The adoption of advanced data analytics and machine learning techniques could revolutionize the analysis of nephrology data, uncovering patterns and insights that are not readily apparent through traditional methods. This approach can enhance the predictive power of the census and other data sources, offering more precise forecasts of disease trends and the impact of interventions.
3. **Enhance data sharing and transparency**: Establishing a framework for increased data sharing and transparency can foster collaboration within the nephrology community and beyond. By making anonymized, aggregated data available to researchers, clinicians, policy makers, industry, and users, the census can stimulate a wider array of initiatives aimed at improving kidney care. This shift requires careful consideration of privacy and ethical guidelines, but promises a richer dataset for improving patient care.
4. **Expand outreach and inclusivity**: Expanding outreach to all Brazilian regions and including minorities and underserved communities will broaden the reach of the BDS, and inclusivity efforts will ensure even greater representation and diversity in the data collected.

## Conclusion

The BDS is a beacon of progress on the nation’s journey towards excellence in kidney care. As we celebrate the multi-decade legacy of the BDS, the community must embrace the opportunity to grow and evolve. By improving methodology, expanding coverage, and leveraging technology, the census can continue to play a pivotal role in shaping the future of nephrology in Brazil, ensuring that every patient’s journey is based on the most comprehensive and insightful data.
